# The Use of a Stably Expressed FRET Biosensor for Determining the Potency of Cancer Drugs

**DOI:** 10.1371/journal.pone.0107010

**Published:** 2014-09-04

**Authors:** William P. Bozza, Xu Di, Kazuyo Takeda, Leslie A. Rivera Rosado, Sarah Pariser, Baolin Zhang

**Affiliations:** 1 Division of Therapeutic Proteins, Office of Biotechnology Products, Center for Drug Evaluation and Research, Food and Drug Administration, Silver Spring, Maryland, United States of America; 2 Microscopy and Imaging Core Facility, Center for Biologics Evaluation and Research, Food and Drug Administration, Silver Spring, Maryland, United States of America; 3 Brown University, Providence, Rode Island, United States of America; University of Wisconsin - Madison, United States of America

## Abstract

Many cancer drugs are intended to kill cancer cells by inducing apoptosis. However, the potency assays used for measuring the bioactivity of these products are generally cell viability assays which do not distinguish between cell death and growth inhibition. Here we describe a cell-based fluorescence resonance energy transfer (FRET) biosensor designed to measure the bioactivity of apoptosis inducing cancer drugs. The biosensor contains cyan fluorescent protein (CFP) linked via caspase 3 and caspase 8 specific cleavage recognition sequences to yellow fluorescent protein (YFP). Upon caspase activation, as in the case of apoptosis induction, the linker is cleaved abolishing the cellular FRET signal. This assay closely reflects the mechanism of action of cancer drugs, in killing cancer cells and therefore can function as a potency test for different cancer drugs. We rigorously demonstrate this through characterization of a class of proteins targeting the death receptors. The one-step assay appears to be superior to other apoptosis-based assays because of its simplicity, convenience, and robustness.

## Introduction

Potency is a measure of the activity of a drug in terms of concentration or amount required to produce a defined biological effect [1]. Therefore, a potency assay should be designed to reflect as much as possible the mechanisms of action of a drug. For oncogenic drugs, which are intended to kill cancer cells, a potency assay would be a measure of the drug’s cytotoxicity in cancer cells. Historically, many chemotherapeutic agents were identified by high throughput screening of compound libraries using cell viability assays with MTT (3-(4,5-dimethylthiazol-2-yl)-2,5-diphenyltetrazolium bromide) or other related dyes [2], [3]. This assay format has been widely adopted as a potency test in the development of cancer drugs. However, cell viability assays cannot distinguish between cell death and growth arrest effects, leaving a caveat in the assessment of the true bioactivity of a cancer drug. As the goal of cancer therapy is to kill cancer cells, it is critical to assess the ability of a drug to induce cancer cell death. The form of cell death most commonly associated with cancer treatment, both *in vivo* and *in vitro* is apoptosis, or programmed cell death.

A hallmark of apoptosis is the activation of caspase proteases, resulting in cleavage of structural proteins and apoptotic body formation [4]. There are two distinct pathways, namely intrinsic and extrinsic, that lead to caspase activation in response to a drug treatment. Classical chemotherapies such as etoposide, compactin, fluorouracil (5-FU), taxol, and camptothecin induce caspase 3 activation in a p53-dependent manner [5]–[7], which often involves mitochondrial alterations. By contrast, a new class of proteins targeting the death receptors expressed on cell surface are under development [8]–[11]. These proteins include the recombinant human tumor necrosis factor (TNF) variants, TNF-related apoptosis-inducing ligand (TRAIL) and death receptor agonistic antibodies. TRAIL binds to death receptor 4 and/or 5 (DR4/5) and subsequently induces the assembly of a death-inducing signaling complex (DISC) containing adaptor protein Fas-Associated protein with Death Domain (FADD) and pro-caspase 8. Within the DISC, pro-caspase 8 becomes activated by self-cleavage and is released into the cytosol wherein it activates caspase 3. In some cell types, the apoptotic signaling is further amplified *via* the mitochondria as a result of the translocation of caspase 8 mediated cleavage of Bid (BH3 interacting-domain protein). Thus, the potency of a cancer drug can be assessed by measuring the magnitude of caspase activity in treated cells. One approach in measuring caspase activity has been the use of fluorescent probes containing caspase substrates, displaying changes in fluorescence intensity upon caspase activation [12]–[16]. Alternatively, active caspases can be detected by immunoblot analysis using antibodies specific to the cleaved form of the individual enzyme [14], [17]–[19]. However, these assays may be associated with large variability due to complex operation procedures. In this study, we have developed a cell-based FRET assay for testing the potency of cancer products. The FRET probe contains recognition sequences for both caspase 3 and caspase 8 and was found to be sensitive towards cancer drugs that act through intrinsic or extrinsic pathways. Notably with the stably expressed FRET probe, the drug-induced response is directly monitored on the treated cells without additional processing steps. Moreover, the FRET assay detects cells undergoing apoptosis with marked sensitivity and experimental simplicity, making it a promising potency assay for the evaluation of cancer drugs.

## Materials and Methods

### Cell lines and reagents

MDA-MB-231 human breast cancer cell line was purchased from the American Type Culture Collection (ATCC, Manassas, VA). A pCMV6-AC plasmid for the expression of a fusion protein (552 amino acids) containing full-length cyan fluorescent protein (CFP) and yellow fluorescent protein (YFP) linked via a caspase recognition sequence was purchased from OriGene (Rockville, MD). The linker between CFP and YFP contains sixteen amino acids, GSGDEVDGGIETDGSG, which can be cleaved by activated caspase 3 at G↓DEVD or caspase 8 at G↓IETD, where ↓ indicates protease cut site. The coding cDNA sequence (1656 base pairs) for the CFP-linker-YFP fusion protein was cloned into the pCMV6-AC vector at Sgf I and Mlu I restriction sites, and the correct construct was verified by nucleotide sequencing. The plasmid was transfected into MB231 cells using Lipofectamine 2000 reagent per the manufacturer’s instruction (Invitrogen, Carlsbad, CA). Transfected cells were selected against neomycin (G418) at 1 mg/ml (Corning Cellgro, Manassas, VA). G418 resistant clones were picked using cloning cylinders (Millipore, Temecula, CA) and tested for fluorescence intensity and protein expression levels. The stable clone with the highest expression levels of the CFP-linker-YFP probe, referred as MB231_CFP-YFP, was used in the following assays. Cells were maintained in DMEM/F-12 50/50 media supplemented with 5% FBS, 4 mM L-glutamine, 1 mM sodium pyruvate, and 0.4 mg/ml G418 sulfate. Parental MB231 cells were cultured in the same media without the addition of G418 sulfate. Both recombinant human TRAIL (rhTRAIL, Cat #375-TEC) and an agonistic monoclonal antibody (Cat #MAB631) specific to the death receptor 5 were purchased from R&D systems (Minneapolis, MN). The rhTRAIL exists as a homotrimer, where each monomer consists of amino acids 114–281 of the native extracellular domain of human TRAIL. Staurosporine was purchased from ApexBio (Boston, MA). Camptothecin and etoposide were purchased from Sigma (St. Louis, MO). 5-FU was purchased from Enzo Life Sciences (Farmingdale, NY). Compactin was purchased from Santa Cruz (Dallas, Texas). Taxol was purchased from Calbiochem (La Jolla, CA).

### Cell viability assay

Cell viability was determined using a 3-(4,5-dimethylthiazol-2-yl)-2,5-diphenyltetrazolium bromide (MTT) colorimetric assay as previously described [20]. Briefly, 1.0–2.0×10^4^ cells were plated in each well of 96 well microplates. Cells were then treated with the cytotoxic agent. After drug treatment, the cell culture media was aspirated and MTT (2 mg/ml in PBS buffer) was then added to each well and was incubated for 2 h at 37°C. Supernatant was then aspirated and insoluble formazan product was dissolved in DMSO. Absorbance for formazan staining was measured at 562 nm using a SpectraMax Plus 384 microplate reader (Molecular Devices, Sunnyvale, CA). Relative OD_562_ for each well was plotted against the log value of concentration of cytotoxic agent in molar. EC_50_ values were determined by fitting the dose-response curve to equation 1, Y = Bottom+(Top-Bottom)/(1+10∧((LogEC_50_-X)*HillSlope)), using the GraphPad Prism software. X is the logarithm of cytotoxic agent concentration in molar and Y is the measured absorbance value. Each data point was repeated in triplicate.

### Flow cytometry

3.0×10^5^ cells were cultured in each well of 6 well plates. Cells were treated with TRAIL or anti-DR5 antibody. Cells were then harvested, stained with Annexin-V-APC (eBioscience, San Diego, CA) and propidium iodide (PI, St Louis, MO), and analyzed using a BD Accuri C6 flow cytometer (BD, Franklin Lakes, NJ) [21].

### Immunoblot analysis

Western blot analysis was performed as previously described [19]. Briefly, cells were lysed using RIPA buffer [0.5 M Tris-HCl (pH 7.4), 1.5 M NaCl, 2.5% deoxycholic acid, 10% NP-40, 10 mM EDTA, and protease inhibitor cocktail] (EMD Millipore, Darmstadt, Germany). Cellular debris was removed by centrifugation and total protein amount was determined by BCA assay (Pierce, Rockford, IL). Equal protein amount (30 µg) was resolved by SDS-PAGE using NuPAGE 4–12% gradient Bis-Tris gels (Invitrogen, Carlsbad, CA) and transferred to PVDF membranes. Antibodies specific to CFP and YFP were from Covance (Gaithersburg, MD), antibodies specific to caspase 8 and caspase 3 were from Cell Signaling (Danvers, MA), and antibodies specific to actin were from Santa Cruz (Dallas, TX). Peroxidase-conjugated anti-mouse, anti-rabbit, or anti-goat IgG antibodies were used as secondary antibodies. Immunoblot detection was visualized by Immobilon Western Chemiluminescent HRP Substrate (Millipore, Billerica, MA) and a LAS-4000 CCD camera system (Fujifilm, Edison, NJ).

### Cell-based FRET cytotoxicity assay

1.8–5.4×10^4^ MB231_CFP-YFP cells were cultured in each well of a black 96 well microplate. After cells were grown overnight, cell culture media was aspirated and PBS buffer containing cytotoxic agent (TRAIL, anti-DR5 antibody, or staurosporine) was added to adhered cells for indicated time points. Due to the requirement of a longer incubation period for camptothecin treatment, cells were treated with DMEM media containing the cytotoxic drug for 48 h before substituting the medium with PBS buffer and allowing incubation to proceed for an additional 24 hours. This procedure was also performed for etoposide, compactin, 5-FU, and taxol. After drug treatment the FRET signal of each sample was determined using a FluoDia T70 fluorescence plate reader (PTI, Edison, NJ). Treated cells were excited using an excitation filter of 425±10 nm. For FRET determination, after background subtraction, emission ratios of YFP (530±5 nm filter) and CFP (486±5 nm filter) were calculated. The FRET values were normalized and plotted against the log value of cytotoxic agent concentration in molar using GraphPad Prism software. EC_50_ values were determined through nonlinear curve fitting to equation 2, Y = 100/(1+10∧((LogEC_50_-X)*Hill Slope)). X is the logarithm of cytotoxic agent concentration in molar and Y is the normalized FRET value. Each data point was repeated in triplicate for a single experiment and each experiment was repeated independently at least twice.

### Biochemical characterization of the FRET probe

To characterize the expression of CFP-linker-YFP probe, the expressed protein was isolated using anti-CFP/YFP antibody. Briefly, 1.2×10^6^ MB231_CFP-YFP cells were harvested and lysed in 1 ml of Pierce immunoprecipitation lysis buffer. 25 µl of anti-CFP/YFP antibody conjugated to sepharose (Abcam, Cambridge, MA) was rotated with debris-free cell lysate for 3 h at 4°C. After washing with lysis buffer, bound CFP- linker-YFP FRET probe was eluted with 50 mM Tris buffer (pH 8.5) containing 1 M NaCl. Immediately after elution the protein was diluted with Tris buffer to yield a final concentration of NaCl of 300 mM. Protein purity was visualized by SDS-PAGE and Coomassie blue staining and by immunoblot analysis using antibodies specific for CFP and YFP (described above).

For *in vitro* FRET assays, immuno-purified CFP-linker-YFP FRET probe was incubated with active caspase 3, caspase 8, or caspase 2 (R & D systems, Minneapolis, MN) for 3 h at 25°C in PBS buffer containing 1 mM DTT using black 96 well microplates. As a control Z-VAD was also added to some reactions. FRET was measured as described above.

### Confocal microscopy

1.5×10^4^ MB231_CFP-YFP cells were cultured on 4 well chambered borosilicate coverglass systems (Nunc, Rochester, NY) for one day before the experiment. Cells were treated with various concentrations of TRAIL (0–25 ng/ml) for 2.5 h before imaging. The cells were imaged with a 63x objective lens and a Zeiss Cell Observer Spinning Disk Confocal Microscope system (Thornwood, NY) with a PECON environmental chamber. FRET efficiency was determined by detecting sensitized emission [22], [23]. Excitation laser lines with a wavelength of 458, 514, and 458 nm were used for donor CFP, acceptor YFP, and FRET channels, respectively. The emission filter for CFP was 485/30 while the emission filter for YFP and FRET was 535/30. Dichroic beam splitter for Yokogawa confocal scan head was RIFT 457/514/647 nm. After obtaining the correction factors for singly expressed CFP and YFP, cell images were acquired for MB231_CFP-YFP cells via CFP, YFP, and FRET channels in an identical manner. Data sets of 9 images were stored as zvi format for future quantitative analysis. Zeiss Axiovision software (ver. 4.8.2) was used for sensitized emission FRET measurement. The FRET efficiency was calculated based on a three-filter system, which is normalized against donor and acceptor intensities, allowing calculation of cross talk and verification of true FRET signal [24], [25]. The value of FRET efficiency was calculated according to Xia’s formula [24]. Three randomly selected images were obtained for each experiment. From these images the FRET efficiency was calculated. Two to three regions of interest (ROI) were placed for a respective cell and ten to fifteen ROIs were subjected to FRET analysis.

## Results

### Generation of a cell-based FRET biosensor

**Figure 1:**
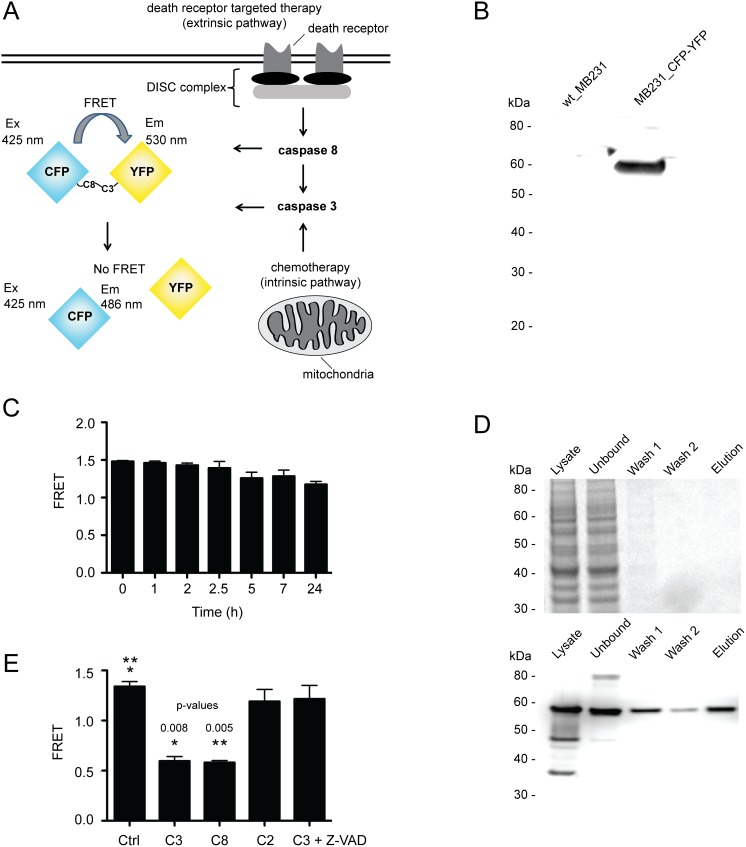
A cell-based FRET biosensor. (A) Schematic view of FRET assay. The FRET probe, CFP-linker-YFP, is expressed as a fusion protein containing CFP and YFP linked via a peptide sequence of 16 amino acids: GSGDEVDGGIETDGSG. The linker region contains recognition sequences for caspase 3 (DEVD) and caspase 8 (IETD), which can be selectively cleaved by its respective enzymes upon induction of intrinsic and/or extrinsic apoptosis pathways. (B) Immunoblot analysis of CFP-linker-YFP FRET probe stably expressed in MB231 cells. (C) Cells expressing CFP- linker-YFP FRET probe produced a significant and stable FRET signal, cells were incubated in PBS buffer for 0–24 h. (D) The CFP- linker-YFP FRET probe was immunopurified from MB231_CFP-YFP cell lysate and visualized by SDS-PAGE and Coomassie Blue staining (top) or by immunoblot analysis (bottom). (E) Changes in FRET were monitored when 100 nM purified CFP- linker-YFP protein was incubated with 100 nM recombinant active caspase 3 (C3), caspase 8 (C8), or caspase 2 (C2) for 3 h. pvalues were determined using a Student’s t-test.

MB231 human breast cancer cells were stably transfected with an expression plasmid encoding CFP-linker-YFP fusion protein (MB231_CFP-YFP), wherein CFP functions as a donor and YFP as an acceptor for fluorescence resonance energy transfer (FRET). The linker contains both caspase 3 and caspase 8 recognition sequences, DEVD and IETD respectively, which are expected to be sensitive to drugs functioning *via* either intrinsic or extrinsic apoptosis pathways. Upon caspase activation following drug treatment, the linker connecting CFP and YFP will be cleaved and subsequently will render a loss of cellular FRET signal. This methodology allows for a powerful FRET biosensor serving as a marker of apoptosis (Figure 1A). The MB231 cell line was chosen as a cell substrate because it has been shown to be fully susceptible to a variety of cytotoxic agents, implying the functional integrity of the apoptosis machinery [3], [18]. Such a cellular platform is expected to be useful for analyzing the potency of different cancer drugs.

We first confirmed expression of the CFP- linker-YFP FRET probe by immunoblot analysis, using a primary antibody that recognizes CFP and YFP. A strong band specific to intact CFP-linker-YFP protein (expected molecular weight 55 kDa) was observed in the lysate of MB231_CFP-YFP cells but not parental cells (Figure 1B). Next we determined whether the MB231_CFP-YFP cells could provide a reasonable FRET signal. In order to eliminate inference from media components, cell culture media was aspirated from MB231_CFP-YFP cells adhered to 96 well microplates. PBS buffer was added to each well and the FRET signal was calculated by taking the ratio between acceptor fluorescence emission (530±5 nm) and donor fluorescence emission (486±5 nm) when the samples were excited at 425±10 nm using a fluorescence plate reader. Indeed under these conditions a significant FRET signal was detected for MB231_CFP- YFP cells (Figure 1C). Notably, the FRET probe was found to be stable in cells submerged in PBS buffer for up to 24 hours (Figure 1C). As a control, untransfected parental MB231 cells were assayed in parallel which did not produce any significant fluorescence signal above background noise when excited at 425±10 nm.

Immunoprecipitation of the CFP-linker-YFP FRET probe was performed to confirm its selectivity for caspase 3 and caspase 8 cleavage. CFP- linker-YFP FRET probe was purified from cell lysate using anti-CFP/YFP antibody conjugated to sepharose beads. Bound FRET probe was eluted using Tris buffer containing 1 M NaCl. Isolated FRET probe was characterized using SDS-PAGE with Coomassie blue staining (Figure 1D, top) and immunoblot analysis (Figure 1D, bottom). To assess caspase specificity, the purified FRET probe was incubated with recombinant forms of active caspase 3, caspase 8, or caspase 2. Changes in FRET were monitored (Figure 1 E). As expected cleavage of the FRET probe was specific for caspase 3 and caspase 8, and led to a pronounced decrease in FRET signal. Consistently, the cleavage of FRET probe was completely abolished when the reaction mixture was incubated with the pan-caspase inhibitor (Z-VAD). By contrast, virtually no cleavage of the FRET probe was detected when CFP- linker-YFP FRET probe was incubated with caspase 2.

### Qualification of the FRET biosensor for use in potency testing for the death receptor targeted cancer therapies

**Figure 2:**
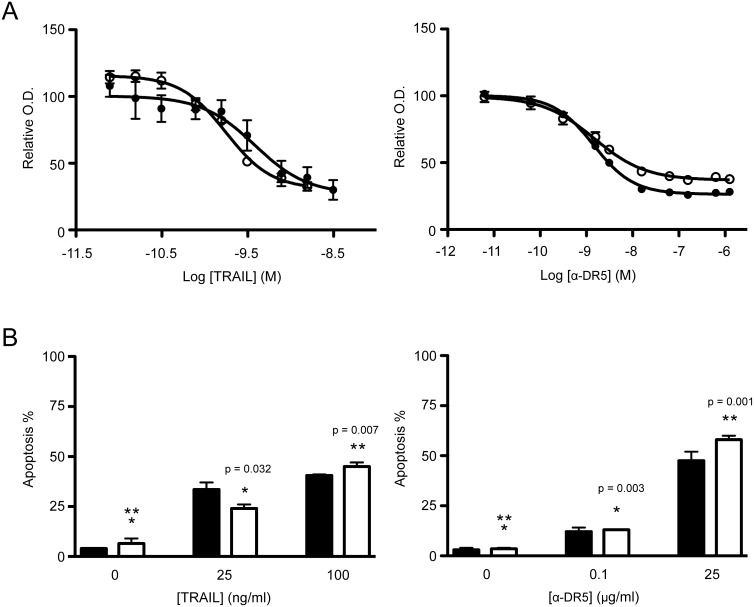
MB231_CFP-YFP cells retain sensitivity to TRAIL and anti-DR5 antibody. (A) Parental MB231 (filled black) and MB231_CFP-YFP (open clear) cells were treated with TRAIL (0–100 ng/ml) for 2.5 h or anti-DR5 antibody (0–200 µg/ml) for 20 h, at the indicated concentrations. Cell viability was determined by MTT assay. (B) Cells were treated as in A and analyzed for apoptosis by flow cytometry after staining with Annexin-V-APC and propidium iodide (PI). p-values were determined using a Student’s t-test. EC_50_ values were determined using nonlinear curve fitting as described in the Materials and Methods section. For each EC_50_ value, 95% confidence intervals reflecting the statistical accuracy are reported in Table 1.

With the stably transfected FRET probe, we first tested its suitability in measuring the potency of a class of proteins that induce apoptosis by targeting the death receptors (DRs). These included recombinant human TNF-related apoptosis inducing ligand (TRAIL) and agonistic antibodies to DR5, which are under development in multiple clinical trials for cancer treatment. These products function through DR4 and/or DR5 expressed on the surface of target cells. Once activated, the intracellular death domain facilitates the assembly of pro-caspase 8/10 and an adaptor protein FADD into a death inducing signaling complex (DISC), leading to a sequential activation of caspase 8 and caspase 3. Thus, these agents serve as excellent probes to test the engineered FRET biosensor. As previously shown for parental MB231 cells [18], MB231_CFP-YFP cells retained a similar susceptibility to TRAIL and anti-DR5 antibody. This was first shown by MTT-based cell viability assays when cells were treated with increasing concentrations of TRAIL or anti-DR5 antibody. A similar dose-response curve and EC_50_ value was obtained for both parental_MB231 cells and MB231_CFP-YFP cells when treated with TRAIL or anti-DR5 antibody (Figure 2A and Table 1). When analyzed for apoptosis induction, both cell lines displayed a similar dose-dependent increase in apoptotic cells after treatment with TRAIL or anti-DR5 antibody (Figure 2B). These data indicate that transfection of FRET biosensor had no effect on the cellular response to DR-targeted therapies. The stable MB231_CFP-YFP cell line appeared to retain intact apoptosis machinery, thus supporting its use as a cell substrate for testing the cytotoxicity of cancer drugs.

**Table 1:** MB231_CFP-YFP dose response EC_50_ values for different cancer products.

	TRAIL	α-DR5 antibody	Staurosporine	Camptothecin
MTT	0.13–0.22 nM	0.93–2.32 nM	46–139 nM	116–156 nM
FRET	0.05–0.08 nM	0.24–0.80 nM	249–990 nM	10–39 nM

All values listed are EC_50_ 95% confidence intervals.

**Figure 3:**
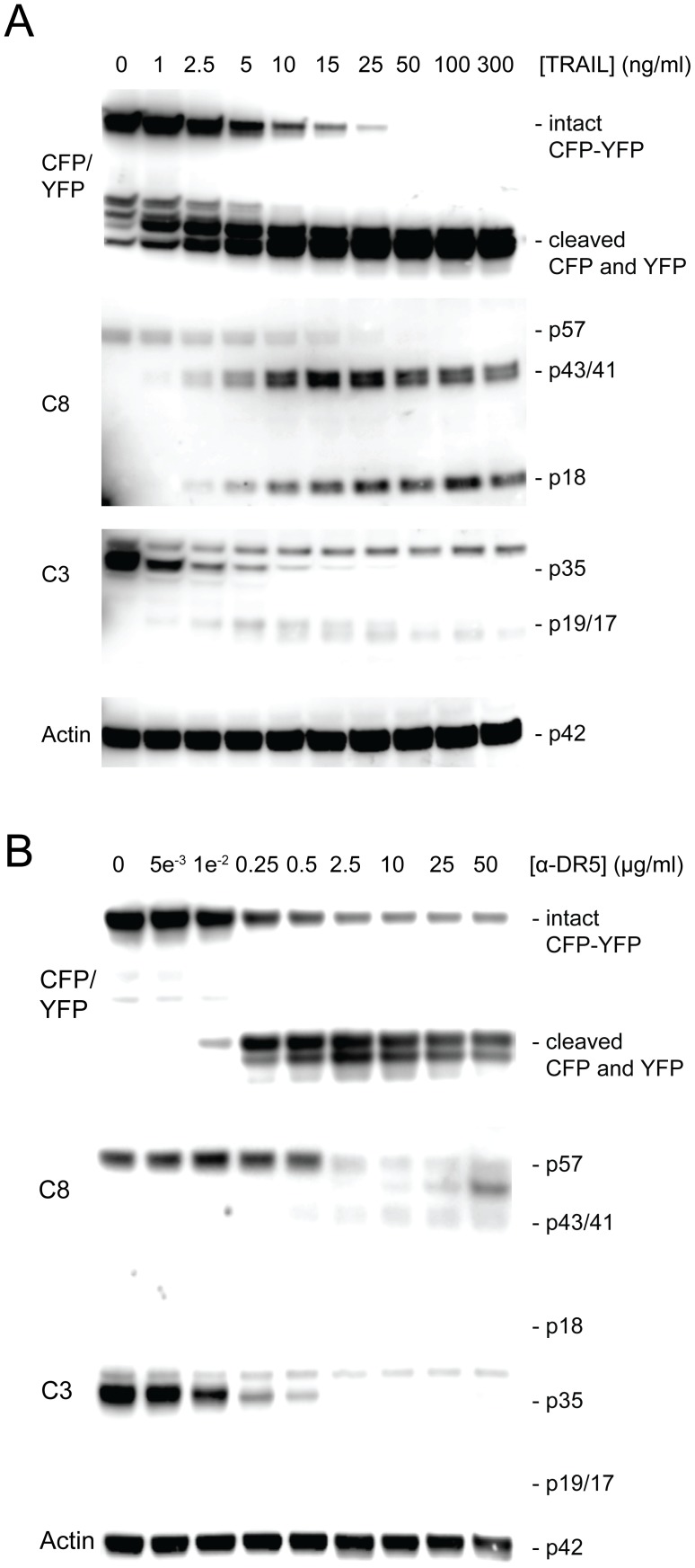
Cleavage of FRET probe is indicative of caspase activation. Cells were treated with (A) 0–300 ng/ml TRAIL for 2.5 hours or (B) 0–50 µg/ml anti-DR5 antibody for 20 h. The resultant cells were analyzed by western blotting using antibodies specific to caspase 3 (C3), caspase 8 (C8), and CFP/YFP.

Next, we assessed cleavage of the FRET probe in response to treatment with TRAIL or anti-DR5 antibody. To this end, MB231_CFP-YFP cells were treated with TRAIL and anti-DR5 antibody. Immunoblot analysis revealed a correlation between caspase activation and cleavage of CFP-linker-YFP FRET probe at all dose levels (Figure 3A and 3B).

**Figure 4:**
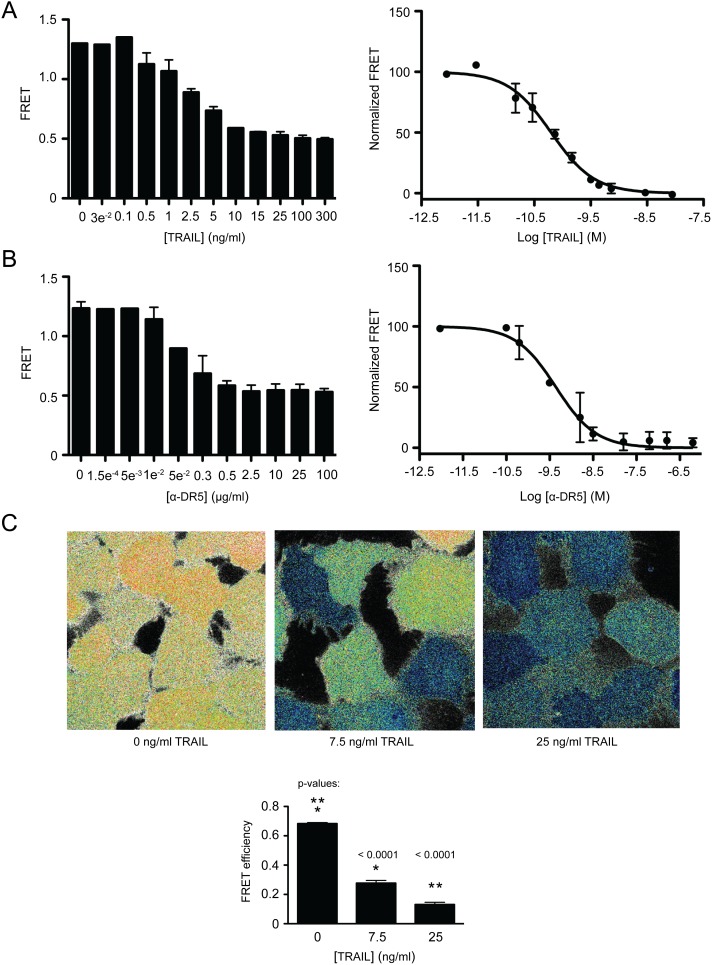
Cell-based FRET biosensor provides a quantifiable response to treatments of death receptor targeted cancer therapies. MB231_CFP-YFP cells were treated with (A) 0–300 ng/ml TRAIL for 2.5 h and (B) 0–100 µg/ml anti-DR5 antibody for 20 h. FRET was calculated and is represented using bar graphs on the left side of the panel. EC_50_ values were determined by nonlinear curve fitting to normalized FRET values plotted against the log values of TRAIL and anti-DR5 antibody concentration in molar (right side of the panel). (C) Confocal microscopy images showing changes in FRET in MB231_CFP-YFP cells treated with 0–25 ng/ml TRAIL for 2.5 h. A pronounced shift from YFP acceptor emission (yellow) to CFP donor emission (cyan) can be observed for cells treated with increasing concentration of TRAIL, with an excitation setting = 458 nm. Statistical analysis was performed as in Fig. 1 and 2.

For FRET measurement, MB231_CFP-YFP cells were cultured in 96 well plates overnight in DMEM media. Cell culture medium was removed and PBS buffer containing various concentrations of TRAIL or anti-DR5 antibody was added to adhered cells for 2.5 h and 20 h respectively. At the indicated times, FRET signals were directly measured on individual samples with an excitation setting of 425±10 nm and emission settings of 530±5 nm for YFP and 486±5 nm for CFP using a fluorescence plate reader. A dose-dependent decrease in FRET was identified with increasing concentrations of TRAIL and anti-DR5 antibody (Figure 4A and 4B). FRET values were normalized and plotted against the log value of TRAIL and anti-DR5 antibody concentration in molar using GraphPad Prism software (Figure 4A and 4B), yielding EC_50_ values after nonlinear curve fitting to equation 2 (Table 1). Notably, the FRET assay produced EC_50_ values that are consistent with those from conventional assays (MTT, flow cytometry, and immunoblot analysis). Collectively, these data support the use of the cell-based FRET assay for analyzing the bioactivity of DR-targeted therapies. Confocal microscopy was also used to evaluate the change in cellular FRET when MB231_CFP-YFP cells were treated with TRAIL (Figure 4C). A pronounced shift from YFP acceptor emission (yellow) to CFP donor emission (cyan) can be observed for cells treated with increasing concentration of TRAIL with an excitation setting = 458 nm.

### Application of the cell-based FRET assay in assessing the bioactivity of cancer chemotherapies

**Figure 5:**
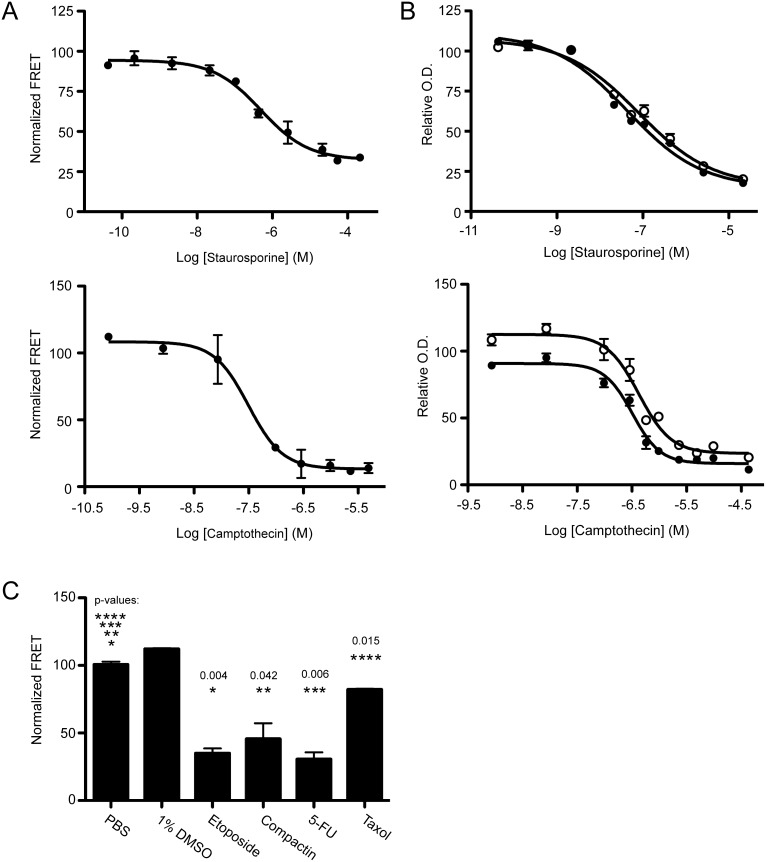
FRET analysis for the bioactivity of chemotherapy agents. (A) MB231_CFP-YFP cells were treated with 0–100 µg/ml staurosporine for 20 h and 0–1.7 µg/ml camptothecin for 72 h. Dose-dependent changes in FRET signals were determined as in Fig. 4. (B) MTT assay was used to evaluate cell viability in response to treatment of parental_MB231 (filled black) and MB231_CFP-YFP (open clear) cells with 0–25 µg/ml staurosporine for 20 h and 0–7.7 µg/ml camptothecin for 72 h, respectively. (C) MB231_CFP-YFP cells were treated with chemotherapy agents (100 µg/ml) for 72 h. FRET measurement and analysis was performed as in Fig. 4. Statistical analysis was performed as in Fig. 1 and 2.

We asked if the engineered FRET probe could be adapted for analyzing small molecule drugs that function through modulation of the intrinsic apoptosis signaling pathways. Many classical chemotherapy drugs fall within this category. We first tested staurosporine and camptothecin, as both are known to be potent inducers of apoptosis in different types of cells [7], [26]. To this end, MB231_CFP-YFP cells were treated with staurosporine or camptothecin. A dose-dependent decrease in FRET was observed with increasing concentrations of each individual agent (Figure 5A). Nonlinear curve fitting produced EC_50_ values that, notably, are comparable with those obtained by MTT analysis (Figure 5B, Table 1). We extended the study to include several other chemotherapy drugs, including etoposide, compactin, 5-FU, and taxol. These drugs function through genotoxic and cytoskeletal stress and lead to subsequent growth inhibition and/or apoptotic cell death, depending on the experimental conditions and the targeted cells [5]–[7]. Consistently, observable FRET signal changes were identified when MB231_CFP-YFP cells were treated with each chemotherapy agent (Figure 5C). However, their ability in inducing caspase 3/8 dependent apoptosis appeared to be much lower than that of TRAIL or anti-DR5 antibody, requiring both high concentration (100 µg/mL) and longer incubation periods (72 h) as compared to the potency of TRAIL (EC50 ∼0.05–0.08 nM) and anti-DR5 (EC50 ∼0.24–0.80 nM). These data demonstrate the ability of the cellular FRET probe in quantifying the apoptosis-inducing activity of both biological and chemotherapeutic agents. By fine-tuning experimental conditions, the FRET assays can be used to directly compare the in vitro bioactivity of different cancer drugs.

## Discussion

We have developed a cell-based FRET biosensor for measuring the activity of apoptosis inducing drugs. Our FRET assay appears to be superior to other commonly used apoptosis assays given its simplicity, stability, convenience, and robustness. We anticipate that given these advantages, the stably expressed FRET probe can be adapted for use in future relevant drug discovery and development studies.

The goal of cancer therapy is to kill cancer cells. Therefore, a potency assay should be designed to reflect the drug’s mechanism of action i.e. inducing apoptosis or other forms of cell death. Apoptosis is a common form of cell death associated with cancer treatment both *in vitro* and *in vivo*. Several methods are available for detecting and measuring apoptosis, including flow cytometry, microscopy, immunoblotting, and caspase substrate-based assays [14], [27]–[29]. These methodologies are powerful research tools in studying apoptosis, but each method is associated with inherent limitations, including but not limited to inability to optimize in a high-throughput manner, variability associated with cell permeability of materials required for a readout signal, requirement of cell lysis, and high background noise [17]. Because of these limitations there is still a need for a standard and robust apoptosis assay. Although several groups have reported cellular expression of a GFP-based fusion protein which can act as a caspase substrate and detect changes in cellular FRET upon induction of apoptosis, detection methods in these instances primarily relied on confocal microscopy and flow cytometry [15], [30]–[33]. While providing valuable information this operating procedure is not ideal for a robust and high throughput potency assay. Notably there are sparse examples reported which utilize a throughput plate format [34], [35]. Our cell-based FRET assay allows for an unprecedented detection of both caspase 3 and caspase 8 activity in a microplate format using a human breast cancer cell line. This caspase combination allows for characterization of any agent that can induce apoptosis through intrinsic or extrinsic pathway or both. Also our methodology has been uniquely adapted to eliminate background auto-fluorescence associated with cell culture media; this helps achieve an improved signal-to-noise ratio and avoids high background subtractions which can complicate FRET calculations. Additionally our work has rigorously focused on the suitability of the described assay in functioning as a potency test for complex protein drugs that target the death receptors while other work have primarily focused on small molecule drug discovery.

One limitation of our assay is related to the availability of molecular target(s) in the MDA-MB-231 cells for a specific drug molecule. For example, MDA-MB-231 cells are known to be deficient in the expression of Her2 receptor thus making the assay unusable for evaluating anti-HER2 monoclonal antibodies that are approved for use in treating breast cancer. This shortcoming, however, could easily be addressed either by expressing the CFP-YFP FRET probe in a HER2 positive cell line or by transfecting HER2 into the MB231_CFP-YFP cell line. In principle, the stable MB231_CFP-YFP cells can serve as a template that can be engineered to express molecular target(s) for a specific drug. Additionally the CFP-YFP FRET probe could be incorporated into other cancer cell lines for testing relevant drugs of interest. It should also be mentioned that the described FRET probe is not suitable for drugs that kill cancer cells through caspase-independent mechanisms or drugs that kill cells through activation of caspases other than caspase 3 or 8, such as caspase 2 mediated apoptosis or necroptosis. Nonetheless, the FRET assay described in this work allows for a one-step detection and measure of apoptosis for treated cells, making it an attractive approach towards a rapid and robust potency assay for cancer drugs or drug candidates that induce caspase-dependent apoptosis. Importantly, the FRET-based platform also allows for distinction between drugs that induce cell death versus those causing growth inhibition, which can in turn guide the selection of combination therapies for a better outcome of cancer treatment.
